# Acute mesenteric ischemia and COVID-19: an integrative review of the literature

**DOI:** 10.1590/0100-6991e-20233334-en

**Published:** 2023-02-17

**Authors:** JULYANNE TEREZA CORDEIRO SILVA, OLIVAL CIRILO LUCENA DA FONSECA

**Affiliations:** 1 - Centro Universitário Maurício de Nassau - Recife - PE - Brasil; 2 - Hospital Universitário Oswaldo Cruz - Recife - PE - Brasil

**Keywords:** Mesenteric Ischemia, COVID-19, SARS-CoV-2, Isquemia Mesentérica, COVID-19, SARS-CoV-2

## Abstract

The novel coronavirus disease 2019 (COVID-19) has spread rapidly around the world after the first cases were reported in December 2019 in China. Despite the prevention of the symptoms presented, extrapulmonary manifestations were identified. In particular, there was an increase in cases of Acute Mesenteric Ischemia (AMI), raising its incidence to 1.9%-3.8% in infected patients. The aim of this study was to investigate the existence of an association between IMA and COVID-19 through the literature. An Integrative Literature Review was carried out. The research question was “mesenteric ischemia in patients with COVID-19: coincidence or association?”. After searching the database and applying the inclusion and exclusion criteria, 44 were selected for analysis. COVID-19 was confirmed by RT-PCR and imaging tests, gastrointestinal manifestations, alterations and primarily tomographic imaging findings were identified. Most patients were accelerated to laparotomy. As explanations include direct endothelial and injury by the binding of the ACE-2 virus, between hyperinflammation and hypercoagulability, dysregulation of the renin-angiotensin-aldosterone system and factors associated with the severity of the virus. IMA is an emergency with high associated morbidity and mortality, these cases may be a consequence mainly of the thromboinflammatory mechanism associated with SARS-CoV-2. An early diagnosis, diagnosis and diagnoses are crucial to clinical treatment; an assessment regime should be considered in accordance with current evidence and guidelines.

## RESUMO

A doença do novo coronavírus 2019 (COVID-19) disseminou-se rapidamente pelo mundo após os primeiros casos serem relatados em dezembro de 2019 na China. Apesar da prevalência dos sintomas respiratórios, manifestações extrapulmonares foram sendo identificadas. Particularmente, houve um aumento de casos de Isquemia Mesentérica Aguda (IMA), elevando sua incidência para 1,9%-3,8% em pacientes infectados. O objetivo deste estudo foi investigar a existência de uma associação entre IMA e a COVID-19 através da literatura. Realizou-se uma Revisão Integrativa da Literatura. A pergunta de pesquisa foi “isquemia mesentérica em pacientes com COVID-19: coincidência ou associação?”. Após a busca na base de dados e aplicação dos critérios de inclusão e exclusão, elegeu-se 44 estudos para análise. A COVID-19 foi confirmada por RT-PCR e exames de imagem, foram identificadas manifestações gastrointestinais, alterações laboratoriais e achados de imagem primordialmente tomográfica. A maioria dos pacientes foi submetida à laparotomia. As explicações incluem lesão endotelial direta pela ligação do vírus ao receptor ECA-2, correlação entre hiperinflamação e hipercoagulabilidade, desregulação do sistema renina-angiotensina-aldosterona e fatores associados à gravidade do quadro. A IMA é uma emergência de alta morbimortalidade associada, nesses casos pode ser consequência principalmente do mecanismo tromboinflamatório associado ao SARS-CoV-2. Um alto nível de suspeita clínica, diagnóstico e tratamento precoces são cruciais diante dessa complicação; um regime de anticoagulação deve ser considerado conforme as evidências e diretrizes vigentes.

## INTRODUCTION

The first cases of pneumonia of unknown etiology were identified in December 2019, in Hubei Province, Wuhan City, China^1^. It was an outbreak of SARS-CoV-2, a new type of coronavirus belonging to the same subgenus as Severe Acute Respiratory Syndrome Coronavirus (SARS-CoV) and Middle East Respiratory Syndrome Coronavirus (MERS-CoV), both responsible for epidemics in 2002 and 2012, respectively^2^. In January 2020, the WHO considered the outbreak to be a Public Health Emergency of International Concern^3^, and due to its rapid spread, in March of the same year, declared the COVID-19 disease a pandemic^4^.

The most common clinical manifestations of COVID-19 are fever, cough, myalgia, fatigue, and dyspnea^5^ ^,^ ^6^. However, although the respiratory tract is the primary target of the etiological agent SARS-CoV-2 and the most prevalent complication is the progression to an Acute Respiratory Distress Syndrome (ARDS), extrapulmonary manifestations are becoming more and more frequent^2^. Abdominal manifestations, in turn, range from 3% to 39%^7^. Whereas in the general population the incidence of mesenteric ischemia is 0.09%-0.2%, in patients affected by COVID-19 it rises to 1.9%-3.8%, with high rates of associated morbidity and mortality^8^ ^,^ ^9^.

Despite being an uncommon cause of abdominal pain, AMI is a surgical emergency that consists of an abrupt interruption of intestinal blood flow. It is subdivided into mesenteric ischemia of non-occlusive and occlusive causes (mesenteric arterial embolism, mesenteric arterial thrombosis, and mesenteric venous thrombosis), whose overall mortality ranges from 50-80%, requiring immediate diagnosis and intervention due to the rapid clinical deterioration of patients^10^ ^-^ ^12^.

Exact answers about a certain thromboinflammatory mechanism triggered by SARS-CoV-2^13^ ^-^ ^15^ that possibly leads to AMI are still scarce. It is postulated that the pathogenesis of mesenteric ischemia secondary to COVID-19 is multifactorial: 1) Endothelial dysfunction resulting from expression of the Angiotensin-Converting Enzyme-2 (ACE-2) receptor, the cellular entry pathway of SARS-CoV-2, not only in alveolar cells, but also in vascular endothelium and intestinal cells^13^ ^,^ ^16^ ^,^ ^17^; 2) State of hypercoagulability directly related to the inflammatory response, with activation and exacerbated expression of prothrombotic factors that lead to the formation of fibrin clots^15^ ^,^ ^16^ ^,^ ^18^ ^-^ ^20^; and 3) Factors related to severe COVID-19 conditions - blood stasis and hemodynamic disorders -, such as stimulation of thrombosis and ischemia^9^ ^,^ ^12^.

In this context, considering the epidemiological impact brought by SARS-CoV-2 and the high rate of complications and mortality of mesenteric ischemia itself, this study aimed to investigate the existence of an association between Acute Mesenteric Ischemia and COVID-19 through an analysis of the currently available literaturel.

## METHODS

We carried out an Integrative Literature Review based on the following steps: identification of the problem with the elaboration of a research question; literature search; evaluation and analysis of data; and presentation of the review with its synthesized results and listed limitations^21^ ^,^ ^22^. The guiding question of the study was “mesenteric ischemia in patients with COVID-19: coincidence or association?”.

We performed the bibliographical research in the PubMed database and the descriptors used were “Mesenteric Ischemia” and “COVID-19”, both previously identified in MeSH and DeCS. We added the Boolean operator “AND” between the two terms, resulting in the combination: “‘Mesenteric Ischemia’ AND COVID-19”. We applied no language restrictions.

### Eligibility criteria

We included original studies, reports, or case series, published between January 2020 and July 2022, in any language, that presented cases of acute mesenteric ischemia with previously or concomitantly diagnosed COVID-19 disease. We excluded articles with duplicate cases, meetings abstracts, studies without full text available, or pre-prints.

**Figure 1:**
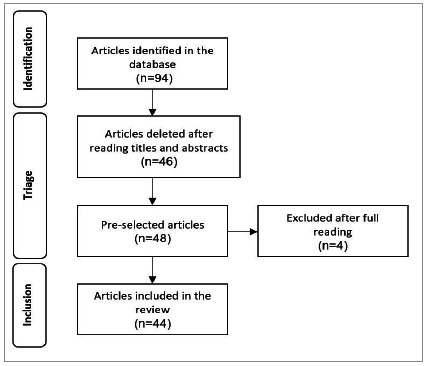

We identified 94 studies in the initial search. After reading the titles and abstracts, we excluded 46 of them and pre-selected the remaining 48 for full text analysis. After complete reading, we excluded four of them, the final sample consisting of 44 studies (Figure 1).

For data collection and organization, we used an adapted instrument, and the critical analysis of the included studies was carried out using the criteria of reduction, display, and comparison of data^21^. Finally, we synthesized and condensed the results in a table (Table 1).

**Tabela 1:** shows the country of each study, number of patients and respective ages, gastrointestinal signs and symptoms, main abdominopelvic imaging findings by computed tomography, conduct, and outcome (hospital discharge or death).

1^st^ Author	Country	n	Age	Gastrointestinal Manifestations	Imaging findings	Conduct	Outcome
Azouz et al.^50^	France	1	56	Abdominal pain, vomiting	Thrombosis in the aortic arch and SMA; dilation and signs of small bowel ischemia	Thrombectomy and laparotomy with resection of the ischemic bowel	NR
Norsa et al.^23^	Italy	1	62	Abdominal pain, vomiting	Thrombosis in the inferior vena cava and SMV; dilation, pneumatosis and signs of ischemia in the small intestine	Laparotomy with resection of the ischemic bowel	Death
Singh et al.^57^	USA	1	82	Abdominal distention and sensitivity	Dilation and pneumatosis in the ascending colon and cecum (without visualization of vessels due to the absence of contrast in the exam)	Laparotomy with resection of the necrotic bowel and ileostomy	Discharge
Sehhat et al.^24^	Will	1	77	Abdominal pain and tenderness	Dilation of loops and thickening of the wall of the small intestine (without visualization of vessels due to the absence of contrast in the exam)	At laparotomy, more than 80cm of ischemic bowel was resected	Death
Rodriguez-Nakamura et al.^51^	Mexico	2	45	Abdominal pain, nausea	Thrombosis in the SMA, signs of ischemia in the distal ileum and cecum	Laparotomy with resection of necrotic bowel	Discharge
			42	Abdominal pain and distention	Thrombosis of the portal and mesenteric veins; mesenteric gas	Laparotomy with resection of necrotic loops, omentectomy and lavage of the cavity due to jejunal perforation	Death
Ucpinar et al.^58^	Turkey	1	82	Abdominal pain and distention; signs of peritonitis	SMA thrombosis; intestinal dilation and pneumatosis; portal and mesenteric venous gas	Died during the preoperative period	Death
Dinoto et al.^59^	Italy	1	84	Abdominal pain and distention; signs of acute abdomen; absent peristalsis	SMA thrombosis; absence of wall enhancement	Endovascular thrombectomy	Death
1^st^ Author	Country	n	Age	Gastrointestinal Manifestations	Imaging findings	Conduct	Outcome
Karna et al.^20^	India	1	61	Abdominal pain and distention; fecaloid vomit	SMA thrombosis with occlusion of the distal ileocolic branch; bowel dilation	Laparotomy with resection of the necrotic bowel and ileostomy	Death
Amaravathi et al.^25^	India	1	45	Abdominal pain	Simultaneous thrombosis of SMA and SMV	Emergency laparotomy and thrombectomy of the SMA; relaparotomy after 48 hours with resection of the necrotic bowel and jejunostomy	NR
Fan et al.^26^	Singapore	1	30	Abdominal pain, vomiting	SMV thrombosis; bowel dilation and obstruction	Laparotomy with resection of necrotic bowel	Discharge
Nasseh et al.^27^	Tunisia	1	68	Abdominal pain, diarrhea	Thickened intestinal wall; terminal obstruction of the ileocolic artery	Unfractionated Heparin and laparoscopy after 3 days	Discharge
Mahruqi et al.^28^	Oman	2	51	NR	Non-Occlusive Mesenteric Ischemia; signs of intestinal hypoperfusion;	Laparotomia foi planejada, mas houve piora clínica e familiares recusaram intervenção cirúrgica	Óbito
			51	Abdominal pain; signs of peritonitis	SMA thrombosis; absence of wall enhancement	Emergency laparotomy with necrotic bowel resection and SMA thrombectomy and 2 relaparotomies at 24h intervals with final jejuno-colic anastomosis	Discharge
Chiu et al.^60^	USA	1	49	Abdominal pain; hematemesis and melena	Distended proximal jejunum with mural thickening	Laparotomy with resection of ischemic bowel	NR
Krothapalli et al.^10^	USA	1	76	Diarrhea and nausea; abdominal pain and distention	SMA and Celiac Trunk thrombosis; intestinal dilation and pneumatosis; absence of wall enhancement	Not a candidate for surgical intervention due to clinical conditions and poor prognosis	Death
Estevez- Cerda et al.^29^	Mexico	1	55	NR	SMA Thrombosis	Laparotomy with resection of the necrotic bowel and jejunostomy	Death
1^st^ Author	Country	n	Age	Gastrointestinal Manifestations	Imaging findings	Conduct	Outcome
Macedo et al.^30^	Brazil	1	53	Abdominal pain, vomiting, belching	Dilated bowel loops and walls thickened by edema; pervious mesenteric vessels	Laparotomy with resection of the ischemic, edematous intestine with zones of stenosis	Discharge
Bannazadeh et al.^31^	USA	1	55	Abdominal pain	SMA Thrombosis	Laparotomy with resection of the necrotic bowel and thrombectomy of the SMA	Discharge
Mir et al.^32^	Iran	2	59	Abdominal pain	Signs of intestinal ischemia and necrosis; perforation, thickened intestinal wall with pneumoperitoneum and free fluid; splenic infarction	Laparotomy revealed necrotic bowel, perforation, and generalized peritonitis.	Death
			60	Abdominal pain	Signs of intestinal ischemia and perforation; air-fluid level and intestinal pneumatosis; renal and splenic infarction	Laparotomy revealed perforation, necrosis of the cecum and ascending colon	Discharge
Aktokmakyan et al.^54^	Turkey	5	61 average	NR	NR	All underwent emergency surgery with a diagnosis of mesenteric ischemia	4/5 Discharge 1/5 Death
Roquetaillade et al.^55^	France, Italy	1	62 media	NR	NR	NR	Death
Moheb et al.^56^	USA	4	60,5 media	NR	NR	Three of the 4 patients underwent surgery	NR
English et al.^33^	England	1	40	Abdominal distension	Hypoperfusion of the small intestine; intestinal pneumatosis	Emergency laparotomy with resection of the ischemic bowel; closure of the abdominal wall after 48h	NR
Pang et al.^34^	Singapore	1	30	Abdominal pain, vomiting	SMV thrombosis, intestinal wall thickening	Conservative management on the 1st admission with LMWH; laparotomy on the 2^nd^ admission	Discharge
Ammar et al.^35^	Pakistan	1	55	Abdominal pain and tenderness, absence of BS	Multiple air-fluid levels	Laparotomy with resection of the gangrenous bowel	Discharge
1^st^ Author	Country	n	Age	Gastrointestinal Manifestations	Imaging findings	Conduct	Outcome
Bianco et al.^36^	Italy	1	59	Abdominal pain, nausea	Air-fluid levels, mesenteric edema, ascites	Laparotomy with resection of the ischemic bowel	Death
Romero et al.^7^	Mexico	1	73	Abdominal pain, nausea and vomiting, signs of peritonitis	Edema and distention of intestinal loops, intestinal pneumatosis,	Laparotomy with resection of the ischemic bowel	Death
Sevella et al.^37^	India	1	44	Abdominal pain and distention, vomiting, constipation, absence of BS	Peritoneal thickening, signs of intestinal ischemia, absent peristalsis, moderate ascites	Laparotomy with resection of the gangrenous bowel	Death
Bagheripour^38^	Iran	1	78	Abdominal pain, tenderness and distention, nausea and vomiting, constipation, absence of BS	Bowel dilation, air-fluid levels, ascites	Diagnostic laparotomy, no intervention performed due to gangrenous extension	Death
Chandrakar et al.^8^	India	2	72	Abdominal pain, vomiting, constipation	Pneumatosis intestinalis, portal venous gas	Laparotomy with resection of gangrenous bowel	Discharge
			70	Abdominal pain, vomiting, constipation	Dilation of intestinal loops, ascites	Laparotomy with resection of gangrenous bowel	Death
Sukegawa et al.^39^	Japan	1	70	Abdominal pain	SMA thrombosis, signs of intestinal ischemia, right renal infarction	Laparotomy with resection of necrotic bowel	Discharge
Marsafi et al.^40^	Morocco	1	33	Abdominal pain and tenderness, absence of BS	Mesenteric, portal, and splenic thrombosis, intestinal wall thickening, submucosal edema, ascites, pneumatosis, and signs of intestinal ischemia	Therapeutic anticoagulation and laparotomy with resection of the ischemic bowel	Death
Costa et al.^9^	Portugal	1	75	Abdominal pain, vomiting, diarrhea	SMA thrombosis, bowel loop dilation	After 2 exploratory laparotomies, it was decided to maintain conservative treatment and proceeded to palliative care	NR
1^st^ Author	Country	n	Age	Gastrointestinal Manifestations	Imaging findings	Conduct	Outcome
Sarkardeh et al.^53^	Iran	24	61,5 median	Abdominal pain	Pneumatosis and intestinal wall thickening, perforation, ascites, pneumoperitoneum	Resection and end-to-end anastomosis of the small intestine was the most common surgical intervention (46%). Terminal ileostomy was performed in 3 patients, Hartmann’s colostomy in 3 others, double-barrel ileostomy in 2 patients. Primary enterorrhaphy was performed in 2. Embolectomy was performed in 1 patient with SMA thrombosis. One patient did not receive any surgical repair due to necrotic extension. Another patient was treated clinically.	62.5% mortality
Hanif et al.^61^	Pakistan	1	20	Abdominal pain, tenderness, and distention	Various levels of fluids	Laparotomy with resection of gangrenous bowel	Discharge
Asghari et al.^41^	Iran	1	51	Abdominal pain, tenderness and distention, nausea	Dilation of bowel loops	Emergency laparotomy on 1^st^ admission, 2 relaparotomies on 2^nd^ admission, with resection of necrotic bowel	Death
Gupta et al.^62^	India	1	55	Abdominal pain, vomiting	SMA Thrombosis	Laparotomy with resection of the gangrenous bowel	Death
Hussein et al.^43^	Saudi Arabia	1	20	Abdominal pain, hematochezia	Mesenteric, portal, splenic, and right hepatic venous thrombosis, intestinal congestion, ascites, pneumatosis, and pneumoperitoneum	At first, mechanical thrombectomy, thrombolytics and anticoagulation. Subsequently, laparotomy with resection of the necrotic intestine.	Discharge
1^st^ Author	Country	n	Age	Gastrointestinal Manifestations	Imaging findings	Conduct	Outcome
Alemán et al.^44^	Ecuador	1	44	Abdominal pain and tenderness	SMV, portal and splenic vein thrombosis	Anticoagulation with LMWH and pain control. He continued with oral anticoagulation for 6 months.	Discharge
Cheung et al.^45^	USA	1	55	Abdominal pain and tenderness, nausea and vomiting, diarrhea, decreased BS	SMA Thrombosis	Therapeutic anticoagulation, SMA thrombectomy, and laparotomy with resection of necrotic bowel	NR
Nada et al.^46^	USA	1	49	Abdominal pain, signs of peritonitis	SMA thrombosis, signs of intestinal ischemia and pneumatosis, pneumoperitoneum, renal infarction	Conservative treatment with LMWH due to advanced intestinal necrosis	Death
Alali et al.^47^	Saudi Arabia	1	35	Abdominal pain and tenderness, vomiting	SMA Thrombosis	Therapeutic anticoagulation, later laparotomy	Discharge
Khaleghi et al.^48^	Iran	1	54	Abdominal pain, tenderness and distention, nausea and vomiting	SMA thrombosis, bowel loop dilation	Laparotomy with resection of necrotic bowel, relaparotomy to assess abnormalities	NR
Posada-Arango et al.^49^	Peru	3	62	Abdominal pain, vomiting	air-fluid levels	At first anticoagulation with UFH, later laparotomy without the possibility of intestinal resection due to necrotic extension	NR
			22	Abdominal pain	SMV thrombosis	Anticoagulation, analgesia, and antibiotic therapy	NR
			65	Abdominal pain and tenderness, hyporexia	Left jejunal artery thrombosis, dilation and signs of intestinal ischemia, splenic infarction	Laparotomy with resection of necrotic bowel	NR
Fransvea et al.^52^	Italy	2	72±7,1 Average	Abdominal pain and distention, nausea and vomiting	SMA thrombosis, signs of intestinal ischemia	Laparotomy with resection of the ischemic bowel, relaparotomy after 48 hours for anastomosis and closure of the abdominal wall	Discharge
1^st^ Author	Country	n	Age	Gastrointestinal Manifestations	Imaging findings	Conduct	Outcome
					SMA thrombosis, signs of intestinal ischemia, splenic and hepatic infarction	Exploratory laparotomy without possibility of intestinal resection due to advanced ischemia	Death

n: number of patients; NR: not reported; SMA: superior mesenteric artery; SMV: superior mesenteric vein; AMI: acute mesenteric ischemia; BS: bowel sounds.

## RESULTS

Among the 44 selected studies, there were 37 case reports/series, two letters, one review with case descriptions, and four other retrospective studies. The mean age of the 45 patients in the reports, series, and letters was 54.9 years, 66.6 30 of whom were male^7^ ^,^ ^8^ ^,^ ^23^ ^-^ ^49^ and three did not have their sex reported^50^ ^-^ ^52^. In an Iranian case series of 24 patients, the median age was 61.5 years and 67% of them were male^53^.

Aktokmakyan et al. presented data from patients who required emergency surgery linked to a positive diagnosis for COVID-19; the average age was 61 years and all were male54. In turn, Roquetaillade et al. conducted a multicenter study in three intensive care departments, analyzing medical records of 20 patients positive for SARS-CoV-2 disease with parallel arterial thromboembolic events; the median age was 62 years (58-70), and the majority (15/20) were male^55^.

A cohort by Moheb et al. compared the incidence of gastrointestinal complications in two groups of patients with ARDS (with and without COVID-19) submitted to the same intensive care protocols; the median age of patients with COVID-19 was 60.5 years (48-71) and 66.5% were male^56^.

In these patients, the diagnosis of SARS-CoV-2 infection was confirmed by the polymerase chain reaction (RT-PCR) technique and/or imaging tests, such as Computed Tomography and X-ray. Peripheral and bilateral consolidations and ground-glass opacities were the most common pulmonary findings^8^ ^,^ ^9^ ^,^ ^20^ ^,^ ^25^ ^,^ ^26^ ^,^ ^28^ ^,^ ^31^ ^,^ ^32^ ^,^ ^34^ ^-^ ^41^ ^,^ ^43^ ^-^ ^49^ ^,^ ^53^ ^,^ ^59^ ^,^ ^61^. Only four reports did not mention the diagnostic method^28^ ^,^ ^52^ ^,^ ^57^ ^,^ ^62^ and there was detection of viral RNA in the intestinal mucosa of a patient through the In Situ Hybridization technique, performed due to the high clinical suspicion associated with a previously negative PCR and absence of inflammatory patterns characteristic of COVID-19 on their chest CT^23^. Fever, cough, and dyspnea were the most reported symptoms^7^ ^,^ ^10^ ^,^ ^20^ ^,^ ^24^ ^,^ ^27^ ^,^ ^28^ ^,^ ^30^ ^-^ ^33^ ^,^ ^37^ ^-^ ^39^ ^,^ ^41^ ^,^ ^47^ ^,^ ^48^ ^,^ ^51^ ^,^ ^53^ ^,^ ^54^ ^,^ ^57^ ^-^ ^61^.

In the studies that cited the gastrointestinal clinical manifestations, the most frequent was abdominal pain with or without other complaints (Table 1), being characterized as intense^7^ ^,^ ^8^ ^,^ ^20^ ^,^ ^24^ ^,^ ^25^ ^,^ ^30^ ^,^ ^31^ ^,^ ^38^ ^,^ ^39^ ^,^ ^41^ ^,^ ^43^ ^,^ ^44^ ^,^ ^51^ ^,^ ^62^, of sudden onset^8^ ^,^ ^31^ ^,^ ^35^ ^,^ ^36^ ^,^ ^38^ ^-^ ^40^ ^,^ ^47^ ^,^ ^58^ ^,^ ^59^, in cramps and without clear triggering factor^34^ ^,^ ^49^ ^,^ ^51^, of diffuse location^8^ ^,^ ^9^ ^,^ ^20^ ^,^ ^28^ ^,^ ^38^ ^,^ ^40^ ^,^ ^41^ ^,^ ^45^ ^,^ ^60^, and epigastric and/or mesogastric^25^ ^-^ ^27^ ^,^ ^49^ ^,^ ^51^.

Ten patients initially admitted with symptoms of COVID-19 were diagnosed with mesenteric ischemia after an interval of 2-27 days of hospitalization^7^ ^,^ ^10^ ^,^ ^20^ ^,^ ^24^ ^,^ ^28^ ^,^ ^33^ ^,^ ^36^ ^,^ ^57^ ^-^ ^59^. Another 19 patients had previously been diagnosed with COVID-19 but returned to the emergency service or required intra/inter-hospital transfer with gastrointestinal complaints^8^ ^,^ ^9^ ^,^ ^26^ ^,^ ^28^ ^,^ ^30^ ^,^ ^31^ ^,^ ^37^ ^,^ ^39^ ^,^ ^43^ ^-^ ^45^ ^,^ ^47^ ^-^ ^49^ ^,^ ^51^ ^,^ ^60^ ^-^ ^62^. In the series with 24 cases by Sarkardeh et al., one of the inclusion criteria was respiratory signs and symptoms and diagnosis of COVID-19 preceding gastrointestinal manifestations or concomitant diagnosis of COVID-19 and intestinal ischemia or perforation; symptoms and signs of ischemia appeared on average seven days (range 2-21) after initial respiratory symptoms^53^.

The positive patients for COVID-19 in the study by Aktokmakyan et al. who arrived at the emergency room receiving a preliminary diagnosis of acute abdomen were subsequently operated on due to mesenteric ischemia^54^. In addition to these, another group resorted to the emergency for gastrointestinal symptoms, with or without the presence of associated respiratory symptoms; in these cases, pneumonia was confirmed after admission^23^ ^,^ ^25^ ^,^ ^27^ ^,^ ^32^ ^,^ ^34^ ^,^ ^35^ ^,^ ^38^ ^,^ ^40^ ^,^ ^41^ ^,^ ^46^ ^,^ ^51^. One patient was admitted for ischemic stroke due to occlusion of the right middle cerebral artery, COVID-19 being confirmed by PCR after suggestive findings in imaging tests and, two days after admission, he developed abdominal pain and vomiting^50^. The other studies did not provide such information.

Hypertension^7^ ^-^ ^10^ ^,^ ^20^ ^,^ ^23^ ^,^ ^24^ ^,^ ^31^ ^,^ ^32^ ^,^ ^39^ ^,^ ^43^ ^,^ ^45^ ^,^ ^49^ ^,^ ^53^ ^,^ ^57^ ^-^ ^60^ and Diabetes Mellitus^7^ ^-^ ^10^ ^,^ ^20^ ^,^ ^23^ ^,^ ^32^ ^,^ ^39^ ^,^ ^57^ ^,^ ^59^ ^,^ ^60^ were the most prevalent comorbidities in these patients. Four of them already had diagnosis of atrial fibrillation^9^ ^,^ ^10^ ^,^ ^39^ ^,^ ^58^: the first had numerous comorbidities and a high atherosclerotic burden, but, as reported, he experienced multiple thrombotic events in a short time interval concomitantly with COVID-19^10^; no finding compatible with embolism was identified by echocardiogram in the second^58^; the third was using Dabigatran and Aspirin^39^, and the fourth, Apixaban^9^.

The others had no significant antecedents and/or showed normality in the tests (electrocardiogram, echocardiogram, CT) in search of arrhythmias, cardioembolic sources, or previous atherosclerosis^24^ ^,^ ^25^ ^,^ ^27^ ^,^ ^28^ ^,^ ^30^ ^,^ ^31^ ^,^ ^34^ ^-^ ^37^ ^,^ ^40^ ^,^ ^41^ ^,^ ^44^ ^,^ ^47^ ^-^ ^50^ ^,^ ^54^ ^,^ ^55^ ^,^ ^59^ ^-^ ^62^.

In the series of cases proposed by Sarkardeh et al., 54% of the patients had no previous comorbidities and the mortality rate due to intestinal ischemia was 62.5%^53^.

Nine studies stated that their patients were on prophylactic anticoagulation with unfractionated heparin (UFH) or low molecular weight (LMWH)^28^ ^,^ ^29^ ^,^ ^31^ ^,^ ^33^ ^,^ ^47^ ^,^ ^53^ ^,^ ^55^ ^,^ ^57^ ^,^ ^58^.

The noticed laboratory abnormalities were leukocytosis^7^ ^-^ ^9^ ^,^ ^20^ ^,^ ^23^ ^,^ ^24^ ^,^ ^27^ ^,^ ^28^ ^,^ ^32^ ^,^ ^36^ ^-^ ^38^ ^,^ ^40^ ^,^ ^41^ ^,^ ^43^ ^-^ ^45^ ^,^ ^48^ ^,^ ^49^ ^,^ ^51^ ^,^ ^53^ ^,^ ^57^ ^-^ ^59^ ^,^ ^61^, elevated or close to the maximum reference values of C-reactive protein (CRP)^9, 10,20,23,24,27,38,41,43,44,46,48,51,53,54,57-59,61^, procalcitonin^7^ ^,^ ^9^ ^,^ ^10^ ^,^ ^57^, lactate dehydrogenase (LDH)^24^ ^,^ ^32^ ^,^ ^37^ ^,^ ^41^ ^,^ ^49^ ^,^ ^53^ ^,^ ^57^ ^,^ ^59^ ^,^ ^61^, ferritin^10^ ^,^ ^25^ ^,^ ^28^ ^,^ ^33^ ^,^ ^44^ ^,^ ^49^ ^,^ ^51^, fibrinogen^26^ ^,^ ^28^ ^,^ ^33^ ^,^ ^34^ ^,^ ^54^ ^,^ ^55^ ^,^ ^57^ ^,^ ^60^ and D-dimer^7^ ^,^ ^10^ ^,^ ^23^ ^,^ ^25^ ^-^ ^28^ ^,^ ^31^ ^,^ ^33^ ^,^ ^34^ ^,^ ^36^ ^-^ ^38^ ^,^ ^40^ ^,^ ^46^ ^,^ ^49^ ^,^ ^51^ ^,^ ^53^ ^-^ ^55^ ^,^ ^57^ ^-^ ^61^, the latter being the most prominent alteration among patients, found in values up to 75 times above the upper limit^23^. The coagulogram revealed alterations in the prothrombin time (PT), international normalized ratio (INR), and/or activated partial thromboplastin time (APTT)^20^ ^,^ ^33^ ^,^ ^37^ ^,^ ^51^ ^,^ ^53^ ^,^ ^54^ ^,^ ^57^ ^,^ ^61^.Some patients also had positive lupus anticoagulant^26^ ^,^ ^34^ and metabolic acidosis^8^ ^,^ ^20^ ^,^ ^24^ ^,^ ^28^, with increased lactate values^8^ ^-^ ^10^ ^,^ ^20^ ^,^ ^28^ ^,^ ^31^ ^,^ ^43^ ^,^ ^58^. All five patients in the study by Aktokmakyan et al.^54^ had impaired clotting time.

Most patients were soon led to emergency laparotomy. Clinical management was chosen for 16 patients: 10 of them later required surgery^9^ ^,^ ^20^ ^,^ ^25^ ^-^ ^27^ ^,^ ^31^ ^,^ ^34^ ^,^ ^45^ ^,^ ^49^ ^,^ ^51^, one of which was laparoscopically^27^ and four did not undergo surgery due to clinical instability and bad prognosis^10^ ^,^ ^28^ ^,^ ^46^ ^,^ ^53^. Scheduled relaparotomies (second-look) were also performed at intervals of 24 to 48 hours^9^ ^,^ ^25^ ^,^ ^28^ ^,^ ^29^ ^,^ ^33^ ^,^ ^41^ ^,^ ^48^ ^,^ ^52^, in addition to revascularization procedures^28^ ^,^ ^31^ ^,^ ^43^ ^,^ ^45^ ^,^ ^47^ ^,^ ^50^ ^,^ ^53^ ^,^ ^59^. One of these patients even underwent an initial exploratory laparotomy and a relaparotomy, but conservative management remained due to severe intraoperative findings and clinical decline^9^.

Histopathological examinations of resected intestinal segments found evidence of areas of necrosis and wall ischemia^7^ ^,^ ^23^ ^,^ ^24^ ^,^ ^26^ ^,^ ^29^ ^,^ ^30^ ^,^ ^32^ ^,^ ^34^ ^,^ ^41^ ^,^ ^53^ ^,^ ^56^ ^,^ ^57^, thrombosis in mesenteric vessels^23^ ^,^ ^24^ ^,^ ^26^ ^,^ ^29^ ^,^ ^31^ ^,^ ^32^ ^,^ ^34^ ^,^ ^41^ ^,^ ^53^ ^,^ ^56^ ^,^ ^57^ ^,^ ^60^, as well as the presence of inflammatory infiltrate and hemorrhagic foci^23^ ^,^ ^24^ ^,^ ^26^ ^,^ ^30^ ^,^ ^53^ ^,^ ^57^. Other findings included severe inflammation in the vascular endothelium^23^ and alterations suggestive of viral inclusion in the cytoplasm of the intestinal glandular epithelial cell^60^. Only 14 studies brought such data.

Moheb et al. noted in their cohort that patients with COVID-19 were more likely to develop gastrointestinal complications compared with those without COVID-19 (74% vs 37%, p<0.001, OR 2.33, 95% CI 1.52 3.63)^56^.

## DISCUSSION

The results of this review are suggestive of an association between Sars-CoV-2 infection and mesenteric ischemia and are in line with what recent evidence has called COVID-19 Associated Coagulopathy (CAC)^13^ ^-^ ^15^. In response to the initial research question, we listed some mechanisms considered to be primarily responsible for the pathogenesis of this acute abdomen condition: a) Vascular endothelial injury and direct intestinal injury; b) Thromboinflammation; c) Dysregulation of the Renin Angiotensin Aldosterone System (RAAS); and d) Factors related to disease severity.

Furthermore, despite the challenges in analyzing and synthesizing different primary sources^21^, we also find it pertinent to address what the literature brings about AMI, since its high morbidity and mortality rate is still the result of the difficulty in early detection and, consequently, late treatment^12^.

### 1) Pathogenesis

#### 1.1 Vascular endothelial injury and direct intestinal injury

As well as the histopathological findings previously described in the results^7^ ^,^ ^23^ ^,^ ^24^ ^,^ ^26^ ^,^ ^29^ ^,^ ^30^ ^,^ ^32^ ^,^ ^34^ ^,^ ^41^ ^,^ ^53^ ^,^ ^56^ ^,^ ^57^, other authors also reported endothelitis mediated by SARS-CoV-2. Varga et al. found evidence of direct viral infection in endothelial cells, presence of inflammatory infiltrate, and apoptotic bodies in vascular sites of different organs, including the small intestine^63^.

Endothelitis induced by COVID-19 occurs due both to direct viral involvement, given the existence of a tropism of SARS-CoV-2 to human endothelium, and to the host’s response to the infection^13^ ^,^ ^15^ ^-^ ^18^ ^,^ ^63^. This association stimulates a procoagulant and hyperinflammatory state, capable of triggering excessive thrombin production and inhibiting fibrinolysis, resulting in vascular dysregulation and consequent organ ischemia^16^ ^,^ ^63^.

SARS-CoV-2 enters the host cell through the interaction between its spike protein and the ACE-2 entry receptor and the coexpression of proteases, such as transmembrane serine 2 (TMPRSS2), is essential for this invasion process^13^ ^,^ ^16^ ^,^ ^17^. Vascular endothelial cells express a large number of ACE-2, which have also been found in tissues such as lung, liver, stomach, intestines, and kidney^13^ ^,^ ^17^. This finding brings an alert to the systemic complications of a virus that does not have only one pulmonary route in the body and justifies the histopathological evidence of tissue damage found in the analyzes of resected intestinal segments.

#### 1.2 Thromboinflammation

Hyperinflammation and hypercoagulability are closely related during SARS-CoV-2 infection: a systemic, exacerbated, and persistent inflammatory response among infected patients, entitled “cytokine storm”^15^ ^,^ ^16^ ^,^ ^19^, is capable of causing a systemic imbalance in physiological anticoagulant pathways, driving the abnormal formation of clots, reduction of fibrinolysis, and even more endothelial injury with recruitment of inflammatory cells and platelet hyperactivation^15^ ^,^ ^18^.

The main cytokines described are interleukin-6 (IL-6) and tumor necrosis factor-α (TNFα) and they have been observed at high levels in patients with COVID-19, especially in the most critical ones^16^ ^,^ ^19^. Complement pathways, in turn, also contribute to the thromboinflammatory mechanism^13^ ^,^ ^15^ ^,^ ^18^.

#### 1.3 Dysregulation of the Renin Angiotensin Aldosterone System

RAAS imbalance during COVID-19 constitutes another pathophysiological mechanism that induces thrombosis, due to the negative regulation of the ACE-2 receptor^18^ ^,^ ^19^. Physiologically, it converts Angiotensin II (ANG-2) into Angiotensin 1-7, an important vasodilator involved in hydroelectrolytic balance and vascular permeability, with antithrombotic and antiproliferative properties^16^. Since Sars-CoV-2 binds to ACE-2 to enter target cells^13^ ^,^ ^16^ ^,^ ^17^, its dysfunction leads to reduced cleavage of ANG-2 into Angiotensin 1-7 and consequent increase in its expression in the organism^16^ ^,^ ^18^ ^,^ ^19^. ANG-2 acts as a potent vasoconstrictor and contributes to the hypercoagulable state. Elevated levels of ANG-2 have been seen in patients with COVID-19^19^.

#### 1.4 Factors related to the severity of COVID-19

Blood stasis at the expense of prolonged immobilization of hospitalized patients, mainly in the ICU^14^, is also a cofactor for thrombosis due to localized hypoxia^13^ ^,^ ^64^, and when associated with endothelial injury and hypercoagulability, composes the well-known Virchow’s Triad, directly related to CAC^14^. Hypoxia in these patients stimulates thrombogenesis not only by increasing blood viscosity, but by directly activating signaling pathways that regulate coagulation (hypoxia-inducible transcription factors) and indirectly by inducing pro-inflammatory mediators^64^ ^,^ ^65^. Finally, hemodynamic instability, such as hypovolemia or sepsis, can lead to Non-Occlusive Mesenteric Ischemia (NOMI) due to reflex splanchnic vasoconstriction^66^ ^-^ ^68^.

### 2) Acute Mesenteric Ischemia

The intestine can withstand a reduction of around 75% in its blood supply for up to 12 hours, due to the wide network of existing mesenteric collaterals^11^ ^,^ ^12^. Beyond bearable, an initially reversible ischemia can progress to necrosis, perforation, peritonitis, and, inevitably, death^12^.

#### 2.1 Mesenteric arterial embolism

In general, arterial embolism is the most common etiology of AMI^11^ ^,^ ^12^. Most emboli are of cardiac origin due to atrial fibrillation (almost 50% of cases), post-infarction, endocarditis, cardiomyopathies, and valvopathies, or even aortic atherosclerotic plaques^11^ ^,^ ^12^ ^,^ ^66^ ^,^ ^67^.

#### 2.2 Mesenteric arterial thrombosis

Arterial thrombosis occurs in approximately 25% of AMI cases^11^ ^,^ ^12^. The main risk factor is the presence of previous chronic atherosclerotic disease, and the others are correlated with it, such as dyslipidemia, hypertension and diabetes^11^ ^,^ ^12^ ^,^ ^66^.

However, during the search for cardioembolic sources or pre-existing atherosclerosis via imaging tests, no finding justified the AMI of the patients in our series, not even those with a previous diagnosis of atrial fibrillation^58^, which, it is worth noting, were on anticoagulation regimen. Thus, even though embolic events may occur, given the clinical diversity and background of patients, the reports (Table 1) strengthen the evidence that SARS-CoV-2 is associated with the acute development of thrombosis and not embolism^58^.

#### 2.3 Non-occlusive mesenteric ischemia

NOMI is seen in 20% of patients with AMI and carries with it a high mortality rate, since it primarily affects the most severe patients^11^ ^,^ ^67^. Its pathogenesis is still poorly understood, usually being a consequence of splanchnic vasoconstriction in response to reduced mesenteric blood flow as a way to ensure perfusion of other vital organs, thus leading to hypoxia and intestinal ischemic injury^11^ ^,^ ^12^ ^,^ ^66^ ^-^ ^68^. Some predisposing factors are hypovolemia, hypotension, sepsis, and use of vasoactive drugs^11^ ^,^ ^12^ ^,^ ^67^.

#### 2.4 Mesenteric venous thrombosis

Mesenteric venous thrombosis is responsible for less than 10% of AMI cases^11^ ^,^ ^12^. However, during this review, it and arterial thrombosis were the most prevalent causes of ischemia concomitant with COVID-19 and, interestingly, there was a case of a patient who simultaneously presented with SMA and SMV thrombosis^25^. Venous obstruction by the thrombus results in intestinal wall edema, increased vascular resistance, and consequent mesenteric ischemia due to reduced arterial blood supply^11^ ^,^ ^12^. The already discussed components of the Virchow’s Triad elucidate the development of venous thrombus. Other causes of hypercoagulability, such as certain hereditary diseases^12^, were not mentioned during patient histories.

### 3) Clinical presentation

The initial scenario for AMI is a nonspecific acute abdomen^11^ ^,^ ^12^. Patients classically present with severe abdominal pain disproportionate to physical examination findings; nausea, vomiting, and diarrhea are common^11^ ^,^ ^12^ ^,^ ^67^ ^,^ ^68^. Abdominal distension and gastrointestinal bleeding with no apparent cause should be taken into account. However, as gastrointestinal symptoms have been frequent in COVID-19 patients in general^16^ and may even precede respiratory symptoms^69^, they become even more nonspecific for AMI. Furthermore, all these clinical manifestations may be masked in those patients sedated in the ICU, the key to an early diagnosis being therefore a high level of clinical suspicion^11^.

### 4) Risk factors

Factors associated with a higher risk of thrombotic complications during COVID-19 are advanced age, male sex, obesity, cardiovascular diseases, hypertension, and diabetes^13^ ^,^ ^16^ ^,^ ^19^. However, as already seen, young patients without significant comorbidities are still subject to complications and high risk of death^70^.

### 5) Complementary exams

#### 5.1 Laboratory tests

Laboratory data, although nonspecific, can be of great value to raise the suspicion of AMI during the course of thromboinflammation mediated by SARS-Cov-2^11^ ^,^ ^68^ ^,^ ^71^. Metabolic acidosis with an elevated lactate level is one of the most common abnormalities^11^ ^,^ ^67^ ^,^ ^71^, but is not specific^71^. Hyperkalemia and hyperphosphatemia are usually late signs of intestinal infarction^67^. Leukocytosis is also frequent^11^ ^,^ ^67^ ^,^ ^69^ ^,^ ^71^ and has been described as a marker of poor prognosis in COVID-19^16^ ^,^ ^18^ ^,^ ^19^ ^,^ ^69^.

D-dimer, a product of fibrin degradation, is also part of the AMI investigation^68^ ^,^ ^71^. Despite being very sensitive and not very specific, its significantly elevated values during COVID-19 denote a high thrombotic risk and are related to disease severity^13^ ^,^ ^15^ ^,^ ^18^ ^,^ ^19^ ^,^ ^72^. Lodigiani et al. demonstrated that D-dimer levels increased substantially during hospitalization of non-surviving patients in a large Italian hospital^73^. In addition to it, CAC is manifested by changes in platelet count, prolonged PT and/or aPTT, and increased fibrinogen, factor VIII, and FvW^13^ ^,^ ^16^ ^,^ ^18^ ^,^ ^70^. The study by Tang et al. observed that abnormalities in these coagulation parameters during COVID-19 are associated with a worse prognosis^74^.

Finally, attention should be paid to the elevation of serum inflammatory markers caused by ischemic damage and the hyperinflammatory state of SARS-CoV-2 infection^16^ ^,^ ^18^ ^,^ ^71^. High levels of CRP, ESR, LDH, ferritin, procalcitonin, and IL-6 are detected^16^ ^,^ ^18^ ^,^ ^19^ ^,^ ^67^ ^,^ ^71^. Positive lupus anticoagulant can also be seen in a number of patients with COVID-19^34^.

### 5.2 Imaging exams

Imaging exams make a crucial contribution to the diagnosis of mesenteric ischemia^12^ ^,^ ^68^. Computed tomographic angiography is the first-line diagnostic modality and should be performed as soon as clinical suspicion arises^11^ ^,^ ^12^ ^,^ ^66^ ^,^ ^75^. In addition to being quick, accessible, and non-invasive^12^ ^,^ ^66^ ^,^ ^75^, it has high sensitivity (89.4%) and specificity (99.5%)^75^. The characteristic findings described in the literature and consistent with those identified during the review are: 1) filling defects in the lumen of mesenteric vessels indicating the existence of thrombi or emboli^12^; 2) reduction or absence of mural enhancement^11^ ^,^ ^12^ ^,^ ^66^; 3) intestinal wall thickening - the most sensitive but nonspecific indicator of ischemia^11^ ^,^ ^12^ ^,^ ^66^ ^,^ ^68^; 4) “halo” or “target” appearance of the intestinal wall due to edema in the submucosal layer interspersed between the mucosa and muscle^11^ ^,^ ^12^ ^,^ ^66^; 5) luminal dilation and a “paper-thin” wall^12^ ^,^ ^66^; 6) intestinal pneumatosis, portomesenteric venous gas, and intraperitoneal free gas are signs of irreversible ischemia^11^ ^,^ ^12^ ^,^ ^66^ ^,^ ^68^; 7) splenomegaly, ascites, and mesenteric fat stranding may also be present^11^ ^,^ ^12^ ^,^ ^66^; 8) In NOMI, these intestinal signs are seen and usually occur in a discontinuous and segmental way, but the mesenteric vessels do not have thromboembolic occlusions^11^ ^,^ ^66^, as reported by two of the included studies^28^ ^,^ ^30^.

Angiography allows a simultaneous diagnostic and therapeutic approach. However, it is currently considered second-line due to its invasive character and low availability in health centers, being destined for endovascular management and cases of NOMI^12^ ^,^ ^66^. Plain X-rays and Ultrasonography are limited in cases of AMI and lack sensitivity and specificity, which is why they are rarely used^11^ ^,^ ^66^ ^-^ ^68^.

### 6) Treatment

Management of AMI involves restoration of mesenteric blood flow, with resection of the necrotic bowel^12^. The initial approach requires fluid resuscitation and aggressive correction of electrolyte abnormalities and acid-base imbalance^11^ ^,^ ^66^ ^,^ ^67^. Anticoagulation with heparin should be started in the absence of contraindications^11^ ^,^ ^66^ and broad-spectrum antibiotics need to be administered due to bacterial translocation and increased risk of sepsis^11^ ^,^ ^12^ ^,^ ^66^ ^,^ ^67^.

Emergency laparotomy is indicated in patients with signs of peritonitis, infarction, or intestinal perforation^11^ ^,^ ^66^. It allows direct visualization of intestinal viability, resection of unviable loops, and early reestablishment of mesenteric blood flow^11^ ^,^ ^66^. However, during this first surgical approach, doubts may remain regarding the ischemic involvement of some segments, thus, planned relaparotomy/ second-look is recommended as part of the management of AMI^11^ ^,^ ^66^ ^,^ ^67^. It is usually performed within 24 to 48 hours of the first approach and, in addition to allowing the resection of initially unnoticed necrotic loops, it also avoids hasty resection of healthy loops by the first approach, reducing the chances of “short bowel syndrome”^11^ ^,^ ^67^.

The endovascular approach can be considered individually when there is no clear evidence of irreversible intestinal ischemia or in combination with conventional open surgery. Revascularization methods vary according to the etiopathogenesis of AMI^11^ ^,^ ^12^ ^,^ ^66^ ^,^ ^67^.

NOMI management is based on correcting the underlying cause of splanchnic vasoconstriction and resection of the necrotic bowel when identified.

### 7) Drug prophylaxis for CAC

Current evidence supports parenteral prophylactic anticoagulation in patients with COVID-19 in the absence of absolute contraindications and especially in the most critical cases^13^ ^,^ ^15^ ^,^ ^16^ ^,^ ^18^ ^,^ ^19^ ^,^ ^70^. The prognosis of patients who use heparin has been better than that of non-users^65^. In addition to its anticoagulant role, a certain anti-inflammatory potential associated with heparin may be able to attenuate the cytokine storm and endothelial damage during COVID-19^19^. Extended thromboprophylaxis is also being advocated post-discharge from COVID-19 in certain cases, but there is still no consensus^13^ ^,^ ^16^ ^,^ ^70^.

### 8) Applicability

Given the severity of AMI and the increase in its incidence in the context of COVID-19, this study can serve as a warning to the multidisciplinary teams that deal with these patients. A general knowledge about the clinical presentation, laboratory alterations, and imaging findings will be able to raise the index of suspicion, allowing diagnosis and treatment as early as possible, to reduce the significant associated morbidity and mortality.

### 9) Study limitations

The results come primarily from reports, case series, and small retrospective studies, which undoubtedly increases the risk of publication bias. In addition, despite the concern to select studies with uniformity as to the description of data, there were differences: not all provided the same laboratory parameters, clear information about anticoagulation, and time of post-discharge follow-up of patients, consequently, no the actual associated mortality rate is known.

Furthermore, COVID-19 is still under investigation and the evidence about it is still scarce, lacking in large studies, which makes it difficult to establish a reliable and well-understood relationship between it and mesenteric ischemia. Obviously, it would be beyond the capacity of this study to cover all the thrombotic potential linked to the new coronavirus. Therefore, answers about the general prevalence of thromboembolic complications resulting from COVID-19, pathophysiology, risk factors, safe use of anticoagulation, and better strategies for diagnosing and managing AMI in these cases should be left to future investigations by large prospective, multicentric, multinational studies.

## CONCLUSION

A process of thromboinflammation and endothelial dysfunction belonging to SARS-CoV-2, together with the severity factors, are responsible for the development of AMI, as well as other thrombotic conditions. A high rate of clinical suspicion followed by early diagnosis and immediate treatment are essential for reducing the mortality associated with this surgical emergency.

Multidisciplinary teams should be attentive to gastrointestinal signs and symptoms during the hospitalization of infected patients, with special attention to those in severe cases admitted to the ICU, and guide them after discharge to seek emergency care in the event of suspicious clinical manifestations. Cell counts, ionograms, and especially coagulation parameters and inflammatory markers need to be monitored and, when AMI is suspected, angiotomography should be performed as soon as possible. The goal of treatment is to reestablish mesenteric blood flow and resect the necrotic bowel. Finally, unless contraindicated, patients with COVID-19 should receive prophylactic anticoagulation as per current guidelines pending consensus through randomized controlled trials.

## References

[1] Organização Pan-Americana da Saúde (2020). Histórico da pandemia de COVID-19.

[2] Cascella M, Rajnik M, Aleem A, Dulebohn SC, di Napoli R (2020). Features, Evaluation, and Treatment of Coronavirus (COVID-19).

[3] Organização Pan-Americana da Saúde (2020). OMS declara emergência de saúde pública de importância internacional por surto de novo coronavírus [Internet].

[4] World Health Oganization (2020). WHO announces COVID-19 outbreak a pandemic.

[5] Huang C, Wang Y, Li X, Ren L, Zhao J, Hu Y (2020). Clinical features of patients infected with 2019 novel coronavirus in Wuhan, China. Lancet.

[6] Wang D, Hu B, Hu C, Zhu F, Liu X, Zhang J (2020). Clinical Characteristics of 138 Hospitalized Patients With 2019 Novel Coronavirus-Infected Pneumonia in Wuhan, China. JAMA.

[7] Romero MDCV, Cárdenas AM, Fuentes AB, Barragán AAS, Gómez DBS, Jiménez MT (2021). Acute mesenteric arterial thrombosis in severe SARS-Co-2 patient A case report and literature review. Int J Surg Case Rep.

[8] Chandrakar S, Sangadala P, Gupta M, Kumar DA, Agarwal A (2022). Acute mesenteric ischemia in COVID-19 Case report and current understanding. Qatar Med J.

[9] Costa F, Nogueira L, Marques S, Torres L, Silva AF (2022). An Improbable Thromboembolic Manifestation of COVID-19 A Case Report. Cureus.

[10] Krothapalli N, Jacob J (2021). A Rare Case of Acute Mesenteric Ischemia in the Setting of COVID-19 Infection. Cureus.

[11] Bala M, Jeffry K, Moore EE, Kluger Y, Biffl W, Gomes CA (2017). Acute mesenteric ischemia guidelines of the World Society of Emergency Surgery. World J Emerg Surg.

[12] Kanasaki S, Furukawa A, Fumoto K, Hamanaka Y, Ota S, Hirose T (2018). Acute Mesenteric Ischemia Multidetector CT Findings and Endovascular Management. Radiographics.

[13] Singhania N, Bansal N, Nimmatoori DP, Ejaz AA, McCullough PA, Singhania G (2020). Current Overview on Hypercoagulability in COVID-19. Am J Cardiovasc Drugs.

[14] Hasan SS, Radford S, Kow CS, Zaidi STR (2020). Venous thromboembolism in critically ill COVID-19 patients receiving prophylactic or therapeutic anticoagulation a systematic review and meta-analysis. J Thromb Thrombolysis.

[15] Connors JM, Levy JH (2020). COVID-19 and its implications for thrombosis and anticoagulation. Blood.

[16] Gupta A, Madhavan MV, Sehgal K, Nair N, Mahajan S, Sehrawat TS (2020). Extrapulmonary manifestations of COVID-19. Nature Medicine.

[17] Qi F, Qian S, Zhang S, Zhang Z (2020). Single cell RNA sequencing of 13 human tissues identify cell types and receptors of human coronaviruses. Biochem Biophys Res Commun.

[18] Abou-Ismail MY, Diamond A, Kapoor S, Arafah Y, Nayak L (2020). The hypercoagulable state in COVID-19 Incidence, pathophysiology, and management. Thromb Res.

[19] Miesbach W, Makris M (2020). COVID-19 Coagulopathy, Risk of Thrombosis, and the Rationale for Anticoagulation. Clin Appl Thromb Hemost.

[20] Karna ST, Panda R, Maurya AP, Kumari S (2020). Superior Mesenteric Artery Thrombosis in COVID-19 Pneumonia an Underestimated Diagnosis-First Case Report in Asia. Indian J Surg.

[21] Whittemore R, Knafl K (2005). The integrative review updated methodology. J Adv Nurs.

[22] Souza MT, Silva MD, Carvalho R (2010). Revisão integrativa o que é e como fazer. Einstein (São Paulo).

[23] Norsa L, Valle C, Morotti D, Bonaffini PA, Indriolo A, Sonzogni A (2020). Intestinal ischemia in the COVID-19 era. Dig Liver Dis.

[24] Sehhat S, Talebzadeh H, Hakamifard A, Melali H, Shabib S, Rahmati A (2020). Acute Mesenteric Ischemia in a Patient with COVID-19 A Case Report. Arch Iran Med.

[25] Amaravathi U, Balamurugan N, Pillai VM, Ayyan SM (2021). Superior Mesenteric Arterial and Venous Thrombosis in COVID-19. J Emerg Med.

[26] Fan BE, Chang CCR, Teo CHY, Yap ES (2020). COVID-19 Coagulopathy with Superior Mesenteric Vein Thrombosis Complicated by an Ischaemic Bowel. Hamostaseologie.

[27] Nasseh S, Trabelsi MM, Oueslati A, Haloui N, Jerraya H, Nouira R (2021). COVID-19 and gastrointestinal symptoms A case report of a Mesenteric Large vessel obstruction. Clin Case Rep.

[28] Mahruqi GA, Stephen E, Abdelhedy I, Wahaibi KA (2021). Our early experience with mesenteric ischemia in COVID-19 positive patients. Ann Vasc Surg.

[29] Estevez-Cerda SC, Saldaña-Rodríguez JA, Alam-Gidi AG, Riojas-Garza A, Rodarte-Shade M, Garza JV (2021). Complicaciones intestinales graves en pacientes SARS-CoV-2 recibiendo manejo protocolizado. Rev Gastroenterol Mex.

[30] Macedo VSO de, Moreira GB, Albuquerque ACF de, Oliveira SC de S, Esmeraldo MA, Barbosa FCB (2021). Late mesenteric ischemia after Sars-Cov-2 infection case report. J Vasc Bras.

[31] Bannazadeh M, Tassiopoulos A, Koullias G (2021). Acute superior mesenteric artery thrombosis seven days after discharge for novel coronavirus pneumonia (NCP). J Vasc Surg Cases Innov Tech.

[32] Mir MZ, Mashhadi A, Jahantigh M, Seyedi SJ (2021). Bowel necrosis associated with COVID-19 pneumonia A report of two cases. Radiol Case Rep.

[33] English W, Banerjee S (2020). Coagulopathy and mesenteric ischaemia in severe SARS-CoV-2 infection. ANZ J Surg.

[34] Pang JHQ, Tang JH, Eugene-Fan B, Lee CL, Low JK (2021). A Peculiar Case of Small Bowel Stricture in a Coronavirus Disease 2019 Patient with Congenital Adhesion Band and Superior Mesenteric Vein Thrombosis. Ann Vasc Surg.

[35] Ammar AS, Naqi SA, Ashraf M, Saleem M, Raza HA, Haider U (2022). A rare case report of short bowel anastomosis after acute mesenteric ischaemia in Covid-19 postive patient. J Pak Med Assoc.

[36] Bianco F, Ranieri AJ, Paterniti G, Pata F, Gallo G (2020). Acute intestinal ischemia in a patient with COVID-19. Tech Coloproctol.

[37] Sevella P, Rallabhandi SS, Jahagirdar V, Kankanala SR, Ginnaram AR, Rama K (2021). Acute Mesenteric Ischemia as an Early Complication of COVID-19. Cureus.

[38] Bagheripour MH, Zakeri MA (2021). Acute Mesenteric Ischemia in a COVID-19 Patient Delay in Referral and Recommendation for Surgery. Case Rep Gastrointest Med.

[39] Sukegawa M, Nishiwada S, Terai T, Kuge H, Koyama F, Nakagawa K (2022). Acute superior mesenteric artery occlusion associated with COVID-19 pneumonia a case report. Surg Case Rep.

[40] Marsafi O, Ijim F, Elkourchi M, Chahbi Z, Adnor S, Wakrim S (2021). Ischémie mésentérique aiguë veineuse chez un jeune sujet COVID-19 positif à propos d'un cas. Pan Afr Med J.

[41] Asghari A, Adeli SH, Vafaeimanesh J, Bagheri H, Riahi M, Mirdamadi M (2021). Intestinal Ischemia Due to Mesenteric Vascular Thrombosis in a Patient with Positive SARS-Cov-2 RNA without Primary Pulmonary Symptom A Case Report. Middle East J Dig Dis.

[42] Gupta PK, Natarajan B, Gupta H, Fang X, Fitzgibbons RJ (2011). Morbidity and mortality after bowel resection for acute mesenteric ischemia. Surgery.

[43] Hussein MH, Alabdaljabar MS, Alfagyh N, Badran M, Alamiri K (2021). Splanchnic venous thrombosis in a nephrotic patient following COVID-19 infection a case report. BMC Nephrol.

[44] Alemán W, Cevallos LC (2021). Subacute mesenteric venous thrombosis secondary to COVID-19 A late thrombotic complication in a nonsevere patient. Radiol Case Rep.

[45] Cheung S, Quiwa JC, Pillai A, Onwu C, Tharayil ZJ, Gupta R (2020). Superior Mesenteric Artery Thrombosis and Acute Intestinal Ischemia as a Consequence of COVID-19 Infection. Am J Case Rep.

[46] Nada A, Shabana A, Elsaadany A, Abdelrahman A, Gaballah AH (2022). Superior mesenteric artery thrombosis and small bowel necrosis An uncommon thromboembolic manifestation in COVID-19 pneumonia. Radiol Case Rep.

[47] Alali AA, Baqais MO, Albishi FM, Alkhamis AI, Alshehri YA, Amri KF (2021). Superior Mesenteric Artery Thrombosis Following Severe COVID-19 Pneumonia. Cureus.

[48] Khaleghi M, Aziz-Ahari A, Rezaeian N, Asadian S, Mounesi Sohi A, Motamedi O (2021). The Valuable Role of Imaging Modalities in the Diagnosis of the Uncommon Presentations of COVID-19 An Educative Case Series. Case Rep Med.

[49] Posada-Arango AM, García-Madrigal J, Echeverri-Isaza S, Alberto-Castrillón G, Martínez D, Gómez AC (2021). Thrombosis in abdominal vessels associated with COVID-19 Infection A report of three cases. Radiol Case Rep.

[50] Azouz E, Yang S, Monnier-Cholley L, Arrivé L (2020). Systemic arterial thrombosis and acute mesenteric ischemia in a patient with COVID-19. Intensive Care Med.

[51] Rodriguez-Nakamura RM, Gonzalez-Calatayud M, Martinez AR Acute mesenteric thrombosis in two patients with COVID-19. Two cases report and literature review. Int J Surg Case Rep.

[52] Fransvea P, Costa G, Pepe G, la Greca A, Magalini S, Puccioni C (2022). Acute intestinal ischemia in patients with COVID-19 single-centre experience and literature review. Eur Rev Med Pharmacol Sci.

[53] Sarkardeh M, Meftah E, Mohammadzadeh N, Koushki J, Sadrzadeh Z (2022). COVID-19 and Intestinal Ischemia: A Multicenter Case Series. Front Med (Lausanne).

[54] Aktokmakyan TV, Tokocin M, Meric S, Celebi F (2021). Is Mesenteric Ischemia In COVID-19 Patients A Surprise. Surg Innov.

[55] Roquetaillade de C, Chousterman BG, Tomasoni D, Zeitouni M, Houdart E, Guedon A (2021). Unusual arterial thrombotic events in Covid-19 patients. Int J Cardiol.

[56] Moheb el M, Naar L, Christensen MA, Kapoen C, Maurer LR, Farhat M (2020). Gastrointestinal Complications in Critically Ill Patients With and Without COVID-19. JAMA.

[57] Singh B, Mechineni A, Kaur P, Ajdir N, Maroules M, Bikkina FS and M (2020). Acute Intestinal Ischemia in a Patient with COVID-19 Infection. Korean J Gastroenterol.

[58] Ucpinar BA U, Sahin C (2020). Superior Mesenteric Artery Thrombosis in a Patient with COVID-19 A Unique Presentation. J Coll Physicians Surg Pak.

[59] Dinoto E, Ferlito F, Marca MA, Mirabella D, Bajardi G, Pecoraro F (2021). Staged acute mesenteric and peripheral ischemia treatment in COVID-19 patient Case report. Int J Surg Case Rep.

[60] Chiu CY, Sarwal A, Mon AM, Tan YE, Shah V (2021). Gastrointestinal COVID-19 related ischemic bowel disease. J Gastroenterol Hepatol.

[61] Hanif M, Ahmad Z, Khan AW, Naz S, Sundas F (2021). COVID-19-Induced Mesenteric Thrombosis. Cureus.

[62] Gupta A, Sharma O, Srikanth K, Mishra R, Tandon A, Rajput D (2022). Review of Mesenteric Ischemia in COVID-19 Patients. Indian J Surg.

[63] Varga Z, Flammer AJ, Steiger P, Haberecker M, Andermatt R, Zinkernagel AS (2020). Endothelial cell infection and endotheliitis in COVID-19. Lancet.

[64] Gupta N, Zhao YY, Evans CE (2019). The stimulation of thrombosis by hypoxia. Thromb Res.

[65] Tang N, Bai H, Chen X, Gong J, Li D, Sun Z (2020). Anticoagulant treatment is associated with decreased mortality in severe coronavirus disease 2019 patients with coagulopathy. J Thromb Haemost.

[66] Olson MC, Fletcher JG, Nagpal P, Froemming AT, Khandelwal A (2019). Mesenteric ischemia what the radiologist needs to know. Cardiovasc Diagn Ther.

[67] Oldenburg WA, Lau LL, Rodenberg TJ, Edmonds HJ, Burger CD (2004). Acute Mesenteric Ischemia A Clinical Review. Arch Intern Med.

[68] Parry AH, Wani AH, Yaseen M (2020). Acute Mesenteric Ischemia in Severe Coronavirus-19 (COVID-19) Possible Mechanisms and Diagnostic Pathway. Acad Radiol.

[69] Acosta S, Ögren M, Sternby NH, Bergqvist D, Björck M (2005). Clinical Implications for the Management of Acute Thromboembolic Occlusion of the Superior Mesenteric Artery Autopsy Findings in 213 Patients. Ann Surg.

[70] Bikdeli B, Madhavan MV, Jimenez D, Chuich T, Dreyfus I, Driggin E (2020). COVID-19 and Thrombotic or Thromboembolic Disease Implications for Prevention, Antithrombotic Therapy, and Follow-Up: JACC State-of-the-Art Review. J Am Coll Cardiol.

[71] Montagnana M, Danese E, Lippi G (2018). Biochemical markers of acute intestinal ischemia possibilities and limitations. Ann Transl Med.

[72] Lippi G, Favaloro EJ (2019). D-dimer is Associated with Severity of Coronavirus. Disease.

[73] Lodigiani C, Lapichino G, Carenzo L, Cecconi M, Ferrazzi P, Sebastian T (2020). Venous and arterial thromboembolic complications in COVID-19 patients admitted to an academic hospital in Milan, Italy. Thromb Res.

[74] Tang N, Li D, Wang X, Sun Z (2020). Abnormal coagulation parameters are associated with poor prognosis in patients with novel coronavirus pneumonia. J Thromb Haemost.

[75] Henes FO, Pickhardt PJ, Herzyk A, Lee SJ, Motosugi U, Derlin T (2017). CT angiography in the setting of suspected acute mesenteric ischemia prevalence of ischemic and alternative diagnoses. Abdom Radiol (NY).

